# Efficacy of CT Guided Pulsed Radiofrequency Treatment for Trigeminal Postherpetic Neuralgia

**DOI:** 10.3389/fnins.2019.00708

**Published:** 2019-07-09

**Authors:** Yuanyuan Ding, Tao Hong, Hongxi Li, Peng Yao, Guangyi Zhao

**Affiliations:** ^1^Department of Pain Management, Shengjing Hospital of China Medical University, Shenyang, China; ^2^Department of Anesthesiology, Shengjing Hospital of China Medical University, Shenyang, China

**Keywords:** trigeminal postherpetic neuralgia, pulsed radiofrequency, trigeminal gasserian ganglion, peripheral nerve, supraorbital nerve, infraorbital nerve, mental nerve

## Abstract

**Objectives:**

Trigeminal postherpetic neuralgia (TPHN) often presents with moderate to severe pain, hyperalgesia, and allodynia. Conventional analgesic treatments are poorly effective, which seriously affects the quality of life. This retrospective study aimed to evaluate the efficacy of pulsed radiofrequency (PRF) for the treatment of TPHN.

**Methods:**

A total of 90 TPHN patients were selected between January 2014 and December 2016 in the Department of Pain Management, Shengjing Hospital, China Medical University. Patients were randomly divided into two groups according to the order of enrollment (*n* = 45 per group): group A, peripheral nerve (supraorbital nerve, infraorbital nerve and mental nerve) PRF; group B, gasserian ganglion PRF. Follow-up assessments of visual analogue scale (VAS) pain assessment, SF-36 health status questionnaire, total efficiency rate, and drug dosage of anticonvulsants and opioid analgesics were performed at time points of 1 week, 1 month, 3 months, 6 months, and 1 year after surgery.

**Results:**

At each postsurgery time point, the VAS decreased, SF-36 (physical and mental components) increased, and drug dosage of anticonvulsants and opioids analgesics decreased in both treatment groups; values at each time point were significantly different from presurgery values (*P* < 0.05). Compared with group A, VAS decreased, SF-36 increased, and dosage of anticonvulsants and opioids analgesics decreased significantly in group B (*P* < 0.05). The total efficiency rates one year after surgery in group A and group B were 68.9 and 86.7%, respectively. The total efficiency rate of group B was statistically higher than that of group A (*P* < 0.05).

**Conclusion:**

PRF relieved TPHN, and gasserian ganglion PRF was more effective than peripheral nerve PRF. The method was effective and improved the quality of life of the patients. PRF is recommended as a treatment for TPHN.

## Introduction

Postherpetic neuralgia (PHN) is a persistent neuropathic pain that remains after a herpes zoster rash has healed. It is the most common complication of herpes zoster infection. The incidence and treatment costs of PHN increase with age (Sicras-Mainar et al., 2012; Duracinsky et al., 2014). Herpes zoster infection of the trigeminal nerve is a high risk factor for PHN (Nalamachu and Morley-Forster, 2012). Trigeminal postherpetic neuralgia (TPHN) often presents with moderate to severe pain, hyperalgesia, and allodynia. Conventional analgesic treatments are ineffective, a consequence that greatly affects the quality of life and increases social burden (Gialloreti et al., 2010; Lovell, 2015). Finding a treatment that can effectively relieve TPHN and improve a patient’s quality of life is imperative.

Trigeminal postherpetic neuralgia is a severe neuropathic pain that occurs when varicella-zoster virus infection affects the trigeminal gasserian ganglion or its divisions. Of the three divisions of the trigeminal nerve, the most commonly involved is the ophthalmic division (V1), which is 20 times more likely to be involved than the other two divisions, the maxillary (V2) division and the mandibular (V3) division (Kaufman, 2008; Nagel et al., 2011). The incidence of ophthalmic herpes zoster is 19% of herpes zoster cases (Wulf et al., 1991), just below the more common incidence of thoracic dermatome involvement (Nagel et al., 2008). Drug treatment for the persistent pain is generally ineffective. Peripheral nerve block is a commonly used treatment that is effective, but there are limitations in its application, and the duration of pain relief is short. Pulsed radiofrequency (PRF) has been used for the treatment of chronic neurological pain. PRF treatment consists of short bursts of high energy current followed by a 480 ms heat dissipation interval, which assures that the temperature of the tissue being treated does not exceed 42°C. Thus, there is no damage to the nerve tissue (Choi et al., 2014), only modulation of nerve function (Lee et al., 2015). There is high incidence of V1 involvement in TPHN. V1 locates in an important anatomic area and great caution must be used in its treatment. The advantage of PRF is that it does not damage the nerve; therefore, there is no contraindication for using PRF for treatment of TPHN. Few studies have been conducted on PRF of the gasserian ganglion as treatment for TPHN.

In this study, PRF treatment of the gasserian ganglion was compared with PRF treatment of peripheral nerves (supraorbital, infraorbital, and mental nerves) for evaluation of its clinical efficacy and satisfaction in TPHN patients.

## Patients and Methods

### Patients

**FIGURE 1:**
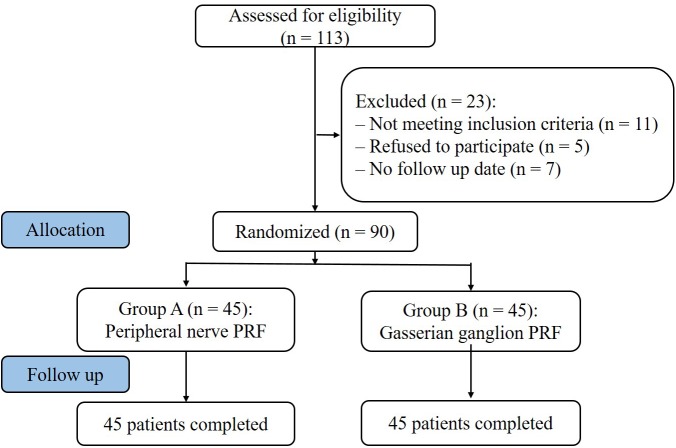
Study flowchart. All 90 patients were included in the treatment. PRF, pulsed radiofrequency.

A total of 90 TPHN patients were selected between January 2014 and December 2016 in the Department of Pain Management, Shengjing Hospital, China Medical University (Figure 1). The patients were randomly divided into two groups according to the order of enrollment (*n* = 45): group A, peripheral nerve PRF (supraorbital nerve, infraorbital nerve and mental nerve); group B, gasserian ganglion PRF. The distribution area of posterior herpes pigments or lesions were unilateral. After the PRF treatment, both groups received steroid injections. Patients were supplemented with anticonvulsants and opioid analgesics. Nutritional supplements that promote nerve recovery and other therapeutic drugs were the same in both groups. The study was approved by the Ethics Committee of Shengjing Hospital, China Medical University. All patients were informed of the risks and complications before surgery, and written informed consents were obtained.

#### Inclusion Criteria

(1) Average pain intensity score (visual analog scale, VAS) ≥5 points in 24 h before enrollment. (2) Skin lesions had healed, but there was still persistent, intense and intractable pain, local skin hyperalgesia, numbness, and sensory abnormalities. (3) Natural course more than 3 months and conservative treatment was ineffective. (4) Age > 50 years old.

#### Exclusion Criteria

(1) Epilepsy, trigeminal neuralgia, or intracranial space-occupying lesion. (2) Hematologic disease or abnormal coagulation, (3) Severe liver and kidney dysfunction or severe cardiopulmonary disease, (4) Drug abuse, (5) Mental illness/unable to cooperate with treatment.

### Surgical Methods

The patient was placed in the supine position. Under CT guidance, the ipsilateral shoulder was positioned and attached to the electrode plate of the radiofrequency apparatus. In group A, PRF of peripheral nerves (supraorbital, infraorbital, and mental nerves) was performed. The point of supraorbital nerve puncture was located in the supraorbital orifice, immediately above the orbital ridge, using the medial line through the pupil as a reference for the supraorbital nerve; The point of infraorbital nerve puncture was located in the infraorbital foramen, can be approximated by having the patient look straight ahead and imagining a line down from the pupil to the inferior border of the infraorbital ridge, bicuspid teeth, and mental foramen; The point of mental nerve puncture was located in the mental foramen, located halfway between the upper (alveolar crest) and the lower edge of the mandible in direct line with the second bicuspid (premolar). In Group B, the Hartel anterior approach to access the gasserian ganglion was performed. The puncture point was positioned 2.5–3 cm from the corner of the mouth. After 0.5% lidocaine was used to anesthetize the ipsilateral-affected side of the mouth, the sterilized radiofrequency needle (21 G, length 100 mm and length of the active tip 5 mm) was guided along the established puncture angle until it reached the site of the foramen ovale. The needle was then guided to the gasserian ganglion. The radio frequency meter was tested to assure coverage of affected area: (1) 50 Hz, 0.1–0.3 V test sensation, to induce pain sensation in the herpes area. (2) 2 Hz, 0.1 V test exercise, jaw tremor will be induced when V3 division is affected. The radiofrequency stimulation tests determined whether the herpes area was covered. The position of the needle tip was adjusted until the movement and sensation determined that the affected area was completely covered and that normal areas were avoided. When the position was satisfactory, each point was treated with 42°C PRF for 300 s, and then 2 ml of an analgesic solution (2% lidocaine 1 ml + vitamin B12 0.5 mg + compound betamethasone 5 mg + normal saline 2 ml) was administered. ceftriaxone sodium was given 30 min before surgery to prevent infection. Vital signs were monitored during surgery. The patient’s reaction was observed during the operation and adjustments made, if needed. The patients were restricted to absolute bed rest for 24 h post treatment.

### Efficacy Evaluation and Follow-Up

Presurgery data were recorded: gender, age, pain duration, presurgery VAS, pain location and divisions affected, and drug dosage of anticonvulsants and opioid analgesics. Follow-up assessments were performed at 1 week, 1 month, 3 months, 6 months, and 1 year after surgery. The non-surgical medical staff used a double-blind method to assess patients at follow-ups.

1. Visual analogue scale: used to evaluate the degree of pain. No pain = 0 points; intense pain = 10 points.
2. SF-36 evaluation (Lam et al., 2005): the quality of life was assessed before and after the operation, including physical and mental status. Physical states include physical function, role physical, bodily pain, and general health. Mental state includes vitality, social function, role emotional, and mental health. The physical component summary (PCS) and mental component summary (MCS) values were averaged.
3. Total efficiency rate: according to subjective symptoms and clinical signs, the efficiency rate was divided into three grades: excellent, effective, and ineffective. Excellent – pain, numbness and hyperalgesia disappeared, and labor levels returned to premorbid levels, effective – pain and numbness were relieved, quality of life improved and ability to work increased, and ineffective – no improvement in symptoms. The total efficiency rate was calculated as the number of patients scoring as “excellent” and “effective” divided by the total number of patients in the group.
4. Drug dosage of anticonvulsants and opioid analgesics: anticonvulsants drugs included carbamazepine, gabapentin, and pregabalin; the opioid analgesic oxycontin was used.

### Statistical Analysis

The data were processed using SPSS18.0 analysis software. The measurement data was first tested for normality, and the single-sample Kolmogorov–Smirnov test was used in non-parametric tests. The normal distribution variables were expressed as mean ± standard deviation ($\bar{x}$ ± SD); One-way analysis of variance was used to compare, and then applied the LSD pairwise comparison. The variables that did not meet the normal distribution were expressed as the median ± interquartile range; The Kruskal–Wallis rank sum test was used in non-parametric tests. The enumeration data were analyzed by chi square test or Fisher’s exact test. *P* < 0.05 was statistically significant.

## Results

### Needle Position on CT Scan and Three-Dimensional Reconstruction

**FIGURE 2:**
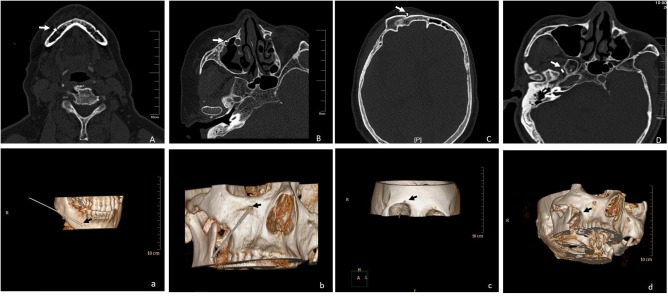
**(A-a)** CT scan and 3D-CT showed the path and position of the needle located in the right mental nerve, shown by the arrow; **(B-b)** CT scan and 3D-CT showed the path and position of the needle located in the right infraorbital nerve, shown by the arrow; **(C-c)** CT scan and 3D-CT showed the path and position of the needle located in the right supraorbital nerve, shown by the arrow; **(D-d)** CT scan and 3D-CT showed the path and position of the needle located in the right foramen ovale, shown by the arrow.

All operations were completed successfully and no serious adverse reactions were observed. On the CT scan plane, the needle tips were located in the peripheral nerve (supraorbital nerve, infraorbital nerve, and mental nerve) or the foramen ovale, respectively. Three-dimensional CT (3D-CT) was performed to further clarify the path and position of the needle (Figure 2).

### Presurgery General Conditions

Presurgery general conditions in the treatment groups included gender, age, pain duration, presurgery VAS, pain location and divisions affected, and presurgery drug dosages of anticonvulsants and opioid analgesics. No significant differences were observed in all general conditions before surgery (*P* > 0.05) (Table 1).

**Table 1:** Presurgery general conditions in patients.

Parameters	Group	
	
	A	B
Patients (*n*)	45	45
Gender (M/F, %)	20 (44.4)/25 (55.6)	22 (48.9)/23(51.1)
Age (year, range)	64.89 ± 8.96 (52–81)	64.76 ± 8.73 (53–82)
Pain duration (m, range)	6.26 ± 2.38 (3–9)	6.31 ± 2.46 (3–10)
Presurgery VAS	6.83 ± 1.41	6.76 ± 1.56
**Pain location – side of face (*n*, %)**		
Right	25 (55.6)	26 (57.8)
Left	20 (44.4)	19 (42.2)
**Divisions affected (*n*, %)**		
V1	5 (11.1)	6 (13.3)
V2	3 (6.7)	3 (6.7)
V3	13 (28.9)	14 (31.1)
V1 V2	10 (22.2)	9 (20.0)
V2 V3	11(24.4)	10 (22.2)
V1 V2 V3	3 (6.7)	3 (6.7)
**Presurgery anticonvulsants drug dosage**		
Carbamazepine (mg/day, *n*)	527.34 ± 73.51 (10)	528.62 ± 72.46 (11)
Gabapentin (g/day, *n*)	2.45 ± 0.35 (21)	2.53 ± 0.29 (19)
Pregabalin (mg/day, *n*)	434.68 ± 71.26 (14)	435.71 ± 72.42 (15)
Presurgery opioids drug dosage		
Oxycontin (mg/day, *n*)	42.43 ± 12.67 (45)	43.08 ± 11.52 (45)


### Visual Analogue Scale Pain Scores Presurgery and Postsurgery

**FIGURE 3:**
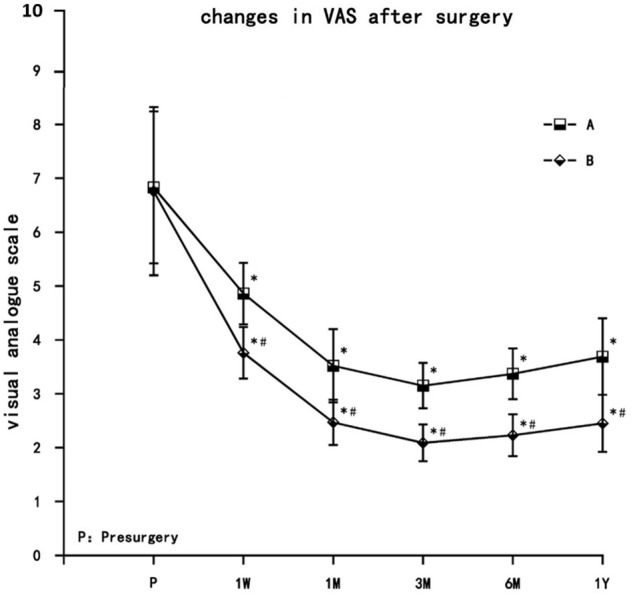
The comparison of VAS pain scores presurgery and postsurgery in both treatment groups. Results are presented as means ± SD. ^∗^Compared to presurgery, *P* < 0.05; #Compared with A group, *P* < 0.05.

Visual analogue scale pain scores decreased in both treatment groups at each postsurgery time point (1 week, 1 month, 3 months, 6 months, and 1 year) and were significantly different from presurgery values (*P* < 0.05). VAS decreased gradually in both groups, with the lowest values reached at 3 months, after which the VAS values increased slightly at 6 months and 1 year but still remained significantly lower than presurgery levels. VAS values in group B were significantly lower than group A at each time point (*P* < 0.05) (Figure 3).

### SF-36 Quality of Life Evaluation Presurgery and Postsurgery

**FIGURE 4:**
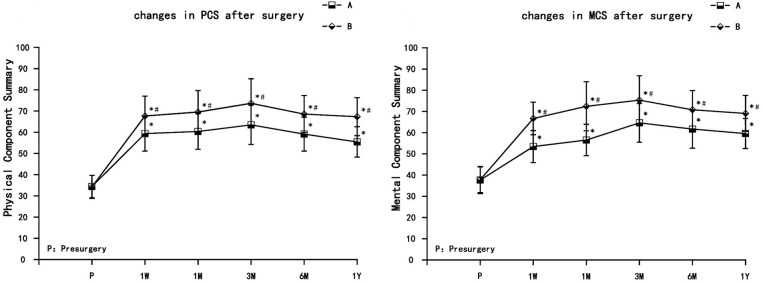
The comparison of quality of life scores (SF-36) presurgery and postsurgery in both treatment groups. PCS, physical component summary; MCS, mental component summary. Results are presented as means ± SD. ^∗^Compared to presurgery, *P* < 0.05; #Compared with group A, *P* < 0.05.

Both groups of patients had different degrees of improvement in quality of life, including physical function, role limitations due to physical health, bodily pain, general health, vitality, social function, role limitations due to emotional health, and mental health. The PCS and MCS increased in both treatment groups at each observation time point postsurgery (1 week, 1 month, 3 months, 6 months, and 1 year), and were significantly different from the preoperative values (*P* < 0.05). PCS and MCS increased gradually in both groups. PCS and MCS values were the highest at 3 months, and decreased thereafter at 6 months and 1 year but still remained significantly higher than presurgery levels. Compared with group A, PCS and MCS increased significantly in group B (*P* < 0.05) (Figure 4).

### The Total Efficiency Rate Postsurgery

The total efficiency rates at 1 year after surgery in group A and group B were 68.9 and 86.7%, respectively. The total effective rate of group B was higher than that of group A, and the difference was statistically significant (*P* < 0.05) (Table 2).

**Table 2:** The comparison of total efficiency rate postsurgery in two groups (%).

Group	*n*	Excellent	Effective	Ineffective	Total efficiency (%)
A	45	20	11	14	68.9
B	45	27	12	6	86.7^∗^


*^∗^Compared with A group, *P* < 0.05.*

### Drug Dosage of Anticonvulsants and Opioid Analgesics Presurgery and Postsurgery

**FIGURE 5:**
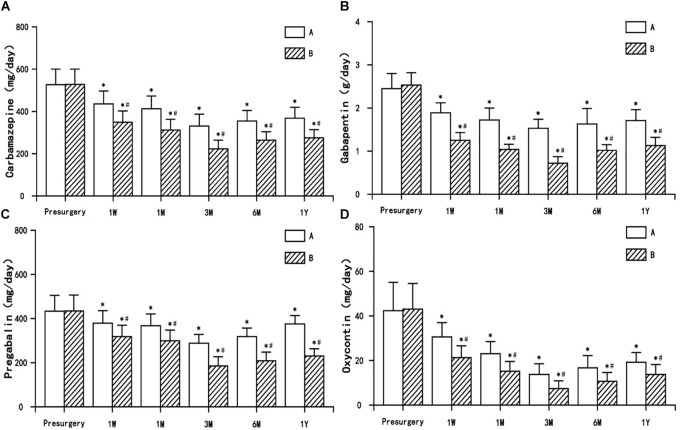
The comparison of the dosage of antiepileptic and opioid drugs presurgery and postsurgery in both treatment groups. **(A)** Carbamazepine, **(B)** Gabapentin, **(C)** Pregabalin, and **(D)** Oxycontin. Results are presented as means ± SD, ^∗^Compared to presurgery, *P* < 0.05; #Compared with A group, *P* < 0.05.

The dosage of anticonvulsants (carbamazepine, gabapentin, and pregabalin) and opioid analgesic (oxycontin) used postsurgery decreased to varying degrees (or were even discontinued) in both treatment groups, and were significantly different from presurgery usage (*P* < 0.05). The decrease in the dosage of anticonvulsants and opioid analgesic in group B was significantly different from the decrease observed in group A (*P* < 0.05) (Figure 5).

## Discussion

Postherpetic neuralgia is a chronic neuropathic pain resulting from re-activation of the herpes zoster virus. The condition is persistent and unresolvable. Its etiology and mechanism are not fully known, and there are no completely effective therapeutic drug or treatment regimens. This study found that after PRF treatment, pain was relieved, quality of life was improved, and the dosage of anticonvulsants and opioid analgesics were reduced in both treatment groups (trigeminal peripheral nerve group and gasserian ganglion group). Gasserian ganglion PRF therapy was superior to peripheral nerve PRF in the relief of pain; the total efficiency rate and patient satisfaction was significantly higher.

The trigeminal nerve is the dominate sensory nerve of the face. After the initial infection of herpes zoster, the virus lies latent in the trigeminal gasserian ganglion. With a decrease in the body’s immune system, the latent herpes zoster virus can reactivate and replicate. Peripheral nerves associated with the infected ganglion will exhibit lesions (Cohrs et al., 2004; Stinson et al., 2017). Severe inflammatory reactions can occur in the invaded ganglia, manifesting as neuronal edema, necrosis, and internal hemorrhage of the nerve, which leads to neuropathic pain (Steain et al., 2014). Trigeminal herpes zoster with symptoms of severe pain has a high probability of developing into PHN (Nagasako et al., 2002). The pain from trigeminal herpes is different from PHN in other areas of the body (Siviero et al., 2011; Rehm et al., 2018); it is more difficult to treat and requires medical attention. Drugs and nerve blocks are the usual courses of treatment to relieve pain. However, incurable pain is often encountered clinically, and conservative treatment alone is ineffective.

Neurodestructive therapy is not recommended for PHN because of an increased chance for neurological damage and aggravation of neuropathic pain (Devulder, 2002). PRF is a minimally invasive treatment of neuropathic pain (Shi and Wu, 2016). As opposed to conventional continuous radiofrequency (CRF) therapy, the temperature of the radiofrequency needle used in PRF does not exceed 42°C, so there is no nerve injury, only modulation of the nerve activity (Perret et al., 2011). PRF of peripheral nerves (supraorbital, infraorbital, and mental nerves) is commonly used (Lim et al., 2013). PRF treatment of the trigeminal gasserian ganglion, however, is able to target the core of the disease. The affected divisions of the trigeminal gasserian ganglion can be identified by RF current stimulation, allowing for treatment closer to the site of the disease and making the treatment more accurate.

The incidence of V1 division involvement is high. Due to the particular anatomy of V1, special care is required during treatment. Therefore, it is necessary to select a treatment method with less side effect. PRF has been shown to cause transient endoneural edema and collagen deposition without structural changes (Podhajsky et al., 2005). The ultrastructural damage of axons, abnormal membrane, and mitochondrial morphology, microtubule and microfilament disintegration were observed under electron microscope (Erdine et al., 2009), but all were reversible, and no contraindication for the treatment of TPHN were found. Therefore, TPHN was treated with gasserian ganglion PRF, and a CT-guided Hartel approach was used to puncture the foramen ovale for accurate positioning. Pain conduction was blocked and the vicious cycle of pain was interrupted temporarily. At the same time, the subsequent local injection of steroids had an anti-inflammation effect, eliminating edema, and improving blood circulation in the area of skin lesions. The functional recovery of damaged nerve terminals and skin receptors was promoted.

The analgesic mechanism of PRF is unclear (Lin et al., 2014). The PRF analgesic effect is not achieved solely through temperature (Podhajsky et al., 2005), but through neuromodulation (Cahana et al., 2006; Rehman et al., 2012). The pain of PHN manifests as burning pain, intermittent sharp pain, paresthesia, thermal, and mechanical hyperalgesia (Garry et al., 2005). The various clinical manifestations of PHN suggest the involvement of the peripheral and central nervous systems, and pathophysiological mechanisms of multiple receptors, pathways, and neurotransmitters (Argoff et al., 2004). It is currently believed that the effect of PRF might be at the microscopic or even subcellular level (Cosman and Cosman, 2005). PRF could selectively act on the fibrous structures of small myelinated Aδ and unmyelinated C pain afferent fibers axons (Jia et al., 2016), inhibit ectopic spontaneous discharge, and interfere with the conduction of nerve impulses. PRF has been shown to regulate the plasticity of synapses in neurons and transiently inhibit induced synaptic activity (Cahana et al., 2003); the effect was reversible and less destructive than CRF treatment. It has also been suggested that the analgesic effect of PRF may be through enhancement of noradrenergic and serotonergic descending inhibitory pathways (Hagiwara et al., 2009). PRF was found to regulate gene expression in a model of nerve injury; expression of GABAB-R1, Na/KATPase, and 5-HT3r anti-inflammatory cytokines were increased, whereas expression of TNF-α and IL-6 pro-inflammatory cytokines were decreased (Vallejo et al., 2013). In another study, the expression of endogenous opioid precursor mRNA (proenkephalin, pro-opiomelanocortin, and prodynorphin), and the corresponding opioid peptide were increased in cultured cells after PRF (Moffett et al., 2012).

Our study showed that PRF effectively relieved the pain of TPHN in both peripheral nerve and gasserian ganglion treatment groups. The neuromodulation effect was relatively slow; the analgesic effect occurred gradually, becoming maximal at the 3 months follow-up. Although VAS increased slightly at the 6 months and 1 year time point, they stayed significantly lower than presurgery levels. The finding that pain was recurring in the latter time points may explain reports that PRF treatment needed to be repeated and combined with drugs and other therapies for optimal analgesia.

In summary, PRF is a recommend treatment to relieve the pain of TPHN. PRF treatment of gasserian ganglion was more effective than treatment of the associated peripheral nerves. The method was effective. PRF reduced VAS, significantly relieved PHN, increased the total efficiency rate, reduced the dosage of anticonvulsants and opioids analgesics, and improved the quality of life for the patients.

## Data Availability

The datasets for this manuscript are not publicly available because this article contains personal information about the patient, so is not publicly available. Requests to access the datasets should be directed to dingyy81@163.com.

## Author Contributions

GZ and PY designed and conducted the study, and contributed to patient recruitment, data collection, and data analysis. HL collected the data. TH analyzed the data. YD prepared the manuscript. All authors approved the final version of the manuscript.

## Conflict of Interest Statement

The authors declare that the research was conducted in the absence of any commercial or financial relationships that could be construed as a potential conflict of interest.
